# Humoral vaccine response and breakthrough infections in kidney transplant recipients during the COVID-19 pandemic: a nationwide cohort study

**DOI:** 10.1016/j.eclinm.2023.102035

**Published:** 2023-06-06

**Authors:** Markus Hovd, Anders Åsberg, Ludvig A. Munthe, Kristian Heldal, Anna V. Reisæter, John T. Vaage, Fridtjof Lund-Johansen, Karsten Midtvedt

**Affiliations:** a Department of Transplantation Medicine, Oslo University Hospital, Norway; b Department of Pharmacy, University of Oslo, Norway; c The Norwegian Renal Registry, Department of Transplantation Medicine, Oslo University Hospital, Norway; d Institute of Clinical Medicine, University of Oslo, Norway; e KG Jebsen Centre for B Cell Malignancies, Institute of Clinical Medicine, University of Oslo, Norway; f Institute of Health and Society, University of Oslo, Norway; g Department of Immunology, Oslo University Hospital, Norway; h ImmunoLingo Convergence Center, Institute of Clinical Medicine, University of Oslo, Norway

**Keywords:** COVID-19, Pandemic, Kidney transplant recipients, Transplantation, Nephrology, Infectious disease, Anti-RBG IgG, Vaccine response, Vaccination, Immunosuppression

## Abstract

**Background:**

Kidney transplant recipients (KTRs) experienced reduced SARS-CoV-2 vaccine response and were at increased risk of severe COVID-19. It is unknown if level of vaccine induced anti-receptor binding domain IgG (anti-RBD IgG) correlates with protection from and survival following COVID-19. We aimed to evaluate the effect of vaccine response on risk of breakthrough infections (BTI) and COVID-19 death in KTRs.

**Methods:**

We performed a nationwide study, examining the competing risk of SARS-CoV-2 infection, COVID-19 related/unrelated death, and vaccine efficacy as assessed by level of anti-RBD IgG response 4–10 weeks after each vaccination. The study included all KTR in Norway alive and with a functioning graft on February 20th, 2020, and events after November 11th, 2022 were right-censored. A pre-pandemic reference-cohort from January 1st 2019 to January 1st 2020 was included to evaluate excess mortality. The study was conducted at Oslo University Hospital, Rikshospitalet, Norway.

**Findings:**

The study included 3607 KTRs (59 [48–70] years) with a functioning graft at February 20th, 2020, who received (median [IQR]) 4 [3–4] vaccines (range 2–6, 99% mRNA). Anti-RBD IgG was measured in 12 701 serum samples provided by 3213 KTRs. Vaccine response was assessed 41 [31–57] days after vaccination. A total of 1090 KTRs were infected with SARS-CoV-2, 1005 (92%) were BTI, and vaccine response did not protect against BTI. The hazard ratio for COVID-19 related death 40 days post-infection was 1.71 (95% CI: 1.14, 2.56) comparing vaccine response levels (≥5 vs. ≥5000 BAU/mL). No excess non-COVID-19 mortality was registered in KTRs surviving SARS-CoV-2 infection compared to a 2019 pre-pandemic reference.

**Interpretation:**

Our findings suggested that SARS-CoV-2 mRNA vaccine response did not predict protection against infection, but prevention of fatal disease progression in KTRs and greater vaccine response further reduced the risk of COVID-19 death. No excess non-COVID-19 mortality was seen during the pandemic.

**Funding:**

10.13039/100016302CEPI and internal funds.

**Research in context**

**Evidence before this study**

Prior to the establishment of our study, a search in PUBMED for studies between 1st Jan 2020 and 1st Dec 2022, using the terms “COVID-19”, “vaccination” and “kidney transplant”, “correlates of protection” revealed a limited number of observational trials on the effect of the novel COVID-19 vaccines on kidney transplant recipients (KTR) in terms of serological correlates, relative risks and protection from COVID-19 related deaths. Studies of SARS-CoV-2 vaccine responses in KTR have established reduced responsiveness in terms of low levels of IgG anti-RBD antibodies that can bind the receptor binding domain of the Spike protein and prevent viral entry.

**Added value of this study**

The main objective was to define the humoral efficacy of vaccination in kidney transplant recipients, the most frequent solid organ transplant recipients, and possible protective effect on SARS-COV-2 infection and COVID-19 related deaths. In this large national analysis of a population with a very high risk of mortality from COVID-19 and limited vaccine response, we find that vaccination had a clear effect on survival. The observed effect was stepwise incremental to vaccine responses assessed by anti-RBD IgG antibody-binding units (BAU) after vaccination. Results demonstrate the importance of assessing vaccine response in highly immunosuppressed patients which may lack the ability to produce a full-scale response comparable to immunocompetent individuals.

**Implications of all the available evidence**

The findings of this study suggest that the new format of mRNA vaccines were successful in reducing the mortality of COVID-19 even in the highly immunosuppressed kidney transplanted population. The evidence highlights the importance of mRNA vaccination of organ transplant recipients. Our results suggest continued vaccination to increase the probability of developing humoral vaccine response, which incrementally correlates with reduced risk of death. Serological response monitoring after vaccination will be useful in guiding clinical decision-making for this vulnerable population. Ultimately, the results from this study have the potential to improve the health outcomes for KTRs and other vulnerable populations in the ongoing COVID-19 pandemic and in future pandemics.

## Introduction

**Fig. 1:**
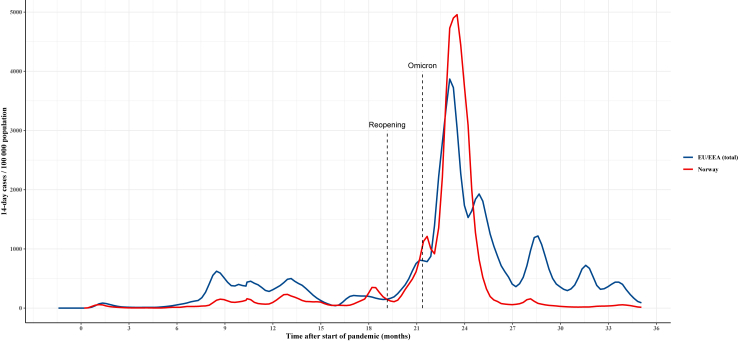
**(SARS-CoV-2 infection rates)**: 14-day notification rate of new infections with SARS-CoV-2 in Norway only (red) and in total for the European Union (EU)/European Economic Area (EAA) member states, Norway included (blue). Data is further described in Methods. “Reopening” refers to removal of national social restrictions to limit the spread of SARS-CoV-2, and “Omicron” refers to the introduction of the Omicron-variant.

Since the onset of the COVID-19 pandemic in December 2019, kidney transplant recipients (KTRs) have been at an increased risk of adverse outcomes if infected with acute respiratory syndrome coronavirus-2 (SARS-CoV-2). A meta-analysis based on 74 studies published before January 18th, 2021, examining 5559 KTR showed that SARS-CoV-2 infection caused 23% mortality and acute kidney injury in 50% of infected patients.^1^ This led to a very strong adherence among KTRs toward governmental recommendations on social distancing and isolation.^2^ Compared to other European countries, Norway was able to keep the overall rate of SARS-CoV-2 infections low, both in the general population and in KTRs (Fig. 1).

With the introduction of SARS-CoV-2 mRNA vaccines, the risk of transmission of the virus and the severity of infection in the general population were successfully reduced, prompting Norway like other countries around the world, to lift social restrictions and re-open society.^3^^,^^4^ However, conventional vaccine strategies against SARS-CoV-2 were shown to be insufficient in KTRs, who remained at risk of a severe course of COVID-19 with a persisting high mortality rate of 5–10%.^5^ As a result, modified vaccine strategies were soon introduced, initially with recommendation of booster-doses.^6^, ^7^, ^8^, ^9^, ^10^, ^11^

However, even after a 4th vaccine dose a considerable proportion of KTRs remain serological non-responders and additional strategies (increased or repeated mRNA vaccine doses, heterologous vaccinations, temporarily withholding immunosuppressive medication at time of vaccination, use of long-lasting monoclonal antibodies) have been investigated, with no clear conclusion so far.^12^, ^13^, ^14^, ^15^, ^16^, ^17^ The objective behind measuring vaccine-induced antibody levels in KTRs has been to provide public health guidelines that can steer risk management, clinical handling and allow personalised recommendations regarding the necessary protective measures, i.e. allowing normal social contact in vaccine responders, and recommend continuous protective measures or further vaccinations in non-responders. However, this has been challenging as the required level of vaccine response to protect against severe disease and death from COVID-19 in KTRs is unknown.

In Norway, KTRs are closely monitored through the Norwegian Renal Registry (NRR), a national registry with a coverage rate of 99.9%. From the start of the pandemic, the NRR (as part of the European Renal Association COVID-19 Database; ERACODA) collected reports from the treating physicians around the country on all SARS-CoV-2 infected KTRs, including time of infection, symptom debut, hospitalisation, admission to the ICU, and COVID-19 related death.^18^^,^^19^ Additionally, from June 2020 the NRR has also performed a national screening initially of COVID-19-induced and eventually of vaccine-induced anti-RBD IgG levels with a novel method at a central laboratory.^20^ KTRs were prioritised along with elderly (≥65 years) and high-risk individuals in the national Norwegian vaccination program.

The primary aim of the present nationwide analysis was to investigate the protective effects of the SARS-CoV-2 humoral vaccine response on deaths due to COVID-19 among KTRs during the pandemic. The secondary aim was to examine any excess mortality in COVID-19 KTR survivors.

## Methods

### Patient data

Patient data were collected from the NRR, including all KTRs, 18 years or older, who were alive and with a functioning graft at the beginning of the pandemic in Norway (February 20th, 2020) in the analysis, and events after November 11th, 2022, were right-censored. Data describing time of infection, symptom debut, hospitalisation, admission to the ICU, and COVID-19 related death was recorded and evaluated by the treating physician at local centres and submitted to the registry. Death dates were also provided to the registry from the national death registry, independent of the reports from local nephrologists, and queries were forwarded to the nephrologist in case of delayed reporting.

In order to compare mortality rate not related to COVID-19 with that in previous years, a reference cohort from 2019 was assessed, which included all consenting KTR alive with a function graft at January 1st, 2019. Events after January 1st, 2020, were right-censored and this cohort had therefore not been exposed to COVID-19. The study was conducted at Oslo University Hospital, Rikshospitalet, Norway.

### Quantification of humoral vaccine response

Antibodies to the receptor-binding domain of SARS-CoV-2 spike protein were measured with a multiplexed bead-based flow cytometric assay as previously described.^20^ For this assay, we consider measurements below 5 BAU/mL as non-specific background. Initially, antibody quantification in serum samples were performed in order to detect sub-clinical infections with SARS-CoV-2 in KTRs. After the introduction of vaccines, here defined as after January 1st, 2021, measurements were performed in order to assess humoral vaccine response. A brief longitudinal overview of when sample collections were performed is available in Figure S1, and patient flow detailing the number of patients with measurements after each vaccine dose is available in Figure S2.

Due to the impaired humoral vaccine response following initial vaccination (dose 1 + 2) all KTR were recommended a booster (dose 3).^21^, ^22^, ^23^ An additional booster (dose 4) was initially prioritised to recipients with less than 2000 BAU/mL following dose 3, but was later offered to all KTRs. In the case of continued low or no vaccine response, additional booster doses were recommended to KTRs.

### Competing risks estimation using a multi-state model

A semi-parametric multi-state model approach combined with a Cox proportional hazard regression model was used to evaluate the competing risk of SARS-CoV-2 infection, COVID-19 related death, and non-COVID-19 related death during the pandemic using the *mstate* and *survival* packages for R 4.2.^24^, ^25^, ^26^ Compared to traditional survival analysis, multi-state models allow patients to transition between a discrete set of states, e.g. from healthy to infected, from infected to death, or from healthy to death. We then estimated the transition intensities, i.e. the cumulative hazard function of transitioning from one state to the other, given the competing outcomes defined in the model.

We developed two structural multi-state models. The first, basic model included four possible states: baseline, infection with SARS-CoV-2, COVID-19 related death, and death by other causes. The multivariate effect of age, sex, time after transplantation and organ source, e.g. living or deceased donor, on transition intensities were evaluated for the basic model only. A schematic of this model is available in Figure S3-A.

Secondly, we developed an extended model to estimate the effect of humoral vaccine response on the risk of infection, death, and COVID-19 related death. Compared to the basic model, this included two additional states: vaccine response, and a separate infection state for patients with vaccine response (Figure S3-B). Patients were considered as with vaccine response at the time of any measured level of anti-RBD IgG above threshold following vaccination, unless already infected with SARS-CoV-2. Models with increasing thresholds for response were compared to evaluate the effectiveness of vaccine response to protect from COVID-19 death. The different threshold levels of anti-RBD IgG required for vaccine response were: 5, 50, 100, 200, 500, 1000, 2000, and 5000 BAU/mL. A supplementary analysis was made for admission to ICU, replacing COVID-19 related death in the extended model. The results for this analysis are available in the Supplementary Data (Figure S4).

### Rate of infection and death in Europe

We obtained data on 14-day rates of infection and death due to COVID-19 in the European Union (EU) and European Economic Agreement (EEA) member states, reported by the European Surveillance System (TESSy) to the European Center for Disease Prevention and Control (ECDC) from the online data portal.^27^ Infection rates were calculated as cases per 100 000 population, with population levels held constant throughout the pandemic. In Norway, infection with SARS-CoV-2 was confirmed with polymerase–chain reaction (PCR) up until fall 2021, from which antigen-based tests were included.

### Ethical considerations

The study was approved according to the research regulations in Norway (Ethical approval numbers: REK Sør-Øst 125548 and 148904), performed according to the Helsinki declaration, and all patients provided informed consent.

### Role of the funding source

This work was funded by Oslo University Hospital, the 10.13039/501100005366University of Oslo, and the 10.13039/100016302Coalition for Epidemic Preparedness Innovations (10.13039/100016302CEPI). Neither funding source were involved in the study design, data collection, analysis and interpretation, writing, or decision to submit the paper for publication. All authors had full access to the data, and accept responsibility for the decision to submit for publication.

## Results

### Patient material

A total of 3607 KTRs (64% male, n = 2314) were included, with median [IQR] age of 59 [48–70] and of which 1364 (38%) had received a kidney from a living donor. The majority of the included KTRs (n = 2733, 76%) were on triple immunosuppresion with calcineurin-inhibitor (CNI; 76% tacrolimus, 24% cyclosporin A), mycophenolic acid, and prednisolon, or dual (n = 389, 11%) immunosuppression consisting of CNI and prednisolone only. A total of 2974 (82%) were prescribed mycophenolic acid (Table S1). The 2019 pre-pandemic reference cohort consisted of 3634 KTR (64% male, n = 2327), aged 59 [48–69] years, of which 1381 (38%) living donor kidney recipient. A total of 160 patients in the reference cohort died between January 1st 2019 and January 1st 2020.

### Vaccination of KTR and humoral vaccine response

**Fig. 2:**
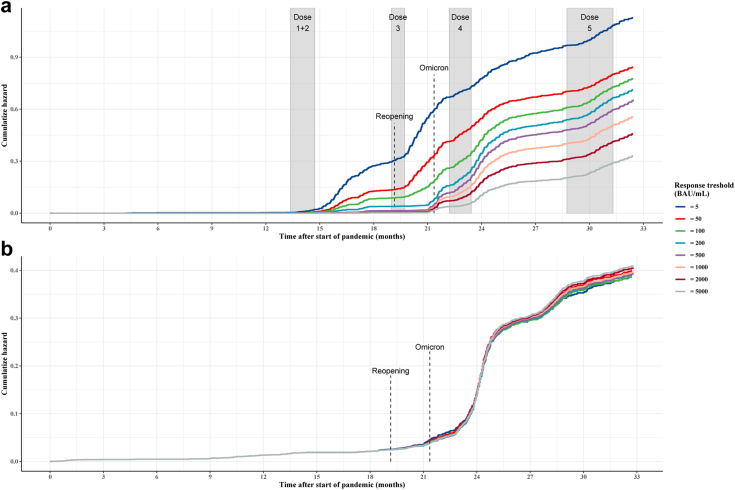
**(Development of vaccine response and risk of infection)**: Likelihood of developing humoral vaccine response (A), here expressed as cumulative hazard since the start of the pandemic and risk of infection with SARS-CoV-2 (B). Dashed lines represent the occurrence of social reopening and detection of the omicron variant, respectively. Gray boxes indicate the inter-quartile range of initial vaccination (dose 1 + 2) and following booster doses.

The national vaccination program rolled out similarly across the country, both for the initial vaccination (dose 1 and 2) and additional booster doses. Due to their immunosuppressed status, KTRs were highly prioritised along with the elderly population (≥65 years). Median time to initial vaccination, consisting of two doses mRNA-vaccine, was 14 months following the start of the pandemic (Table S2). Data following one, two, or three additional booster doses provided during the study period were also included in the analysis, and a total of 3312 (92%) and 3260 (90%) received at least one and two vaccine doses, respectively. A total of 12 702 serum samples (2105 before/10597 after introduction of vaccines) were analysed for anti-RBD IgG in 3213 (89%) patients (2068 before/3068 after vaccination) to detect sub-clinical infections and determine humoral vaccine responses, respectively. Each patient provided 4 [2–5] serum samples, ranging from 1 to 12. Following the initial two vaccinations, response was measured after a median [IQR] of 44 [34–67] days, with subsequent measurements 36 [28–50] days after the 3rd, 29 [26, 35] days after the 4th, and 33 [26, 40] days after the 5th dose. Humoral vaccine response, as evaluated by any quantified anti-RBD IgG measurement above 5 BAU/mL after vaccination (censored for SARS-CoV-2 breakthrough infection), was achieved in 2308 (70%) of vaccinated individuals throughout the study period i.e. 1004 (30%) remained non-responders. In contrast, with a response threshold of 5000 BAU/mL, 1046 (32%) achieved response, leaving 2266 (68%) as non-responders. The likelihood for humoral vaccine response increased with additional booster doses (Fig. 2A).

### Infection with SARS-CoV-2

A total of 1094 (30%) KTRs were infected with SARS-CoV-2 during the study period, of which 1005 (92%) were breakthrough infections (BTI) and occurred after at least two vaccine doses. BTI continued to occur following the 3rd (n = 872, 80%), 4th (n = 573, 52%) and 5th (n = 26, 2%) vaccination dose.

Prior to social re-opening, infection rates were generally low in both the KTR (Fig. 2B) and Norwegian population as a whole (Fig. 1). Following re-opening, under the exposure of the Omicron-variant, infection rates increased dramatically, as was also the case in the rest of Europe. However, there were no differences in risk of infection with SARS-CoV-2 between the different thresholds for vaccine response in KTRs (Fig. 2B). During the study period 74 (7%) of all KTRs infected with SARS-CoV-2 died due to COVID-19, and 80 (7%) were admitted to the ICU following infection. Of all KTR infected with SARS-CoV-2 during the first year of the pandemic i.e. before the introduction of vaccines, a total of 8/42 infected died, indicating a mortality rate of 19%. This later decreased to 10% (24/229) during the second year, with a further reduction to 5% (43/828) during the third year.

### Vaccine response was predictive of survival following infection with SARS-CoV-2

**Fig. 3:**
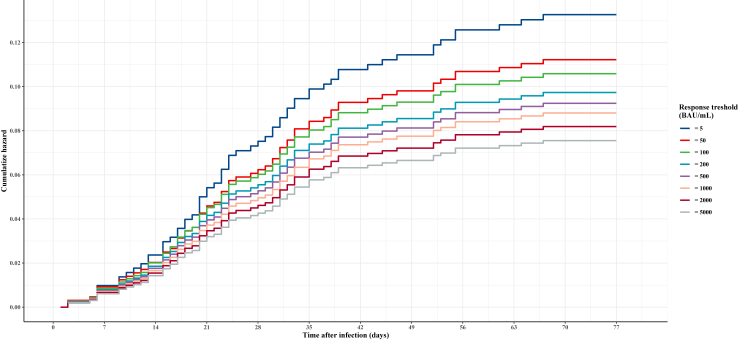
**(Risk of COVID-19 related death in non-responders):** Cumulative hazard for COVID-19 related death in vaccine non-responders after proven infection with SARS-CoV-2, stratified by response thresholds from 5 to 5000 anti-RBD IgG BAU/mL.

Risk of COVID-19 related death decreased with increased humoral vaccine response, as demonstrated by the reduced risk of death in defined responders by increasing the threshold for vaccine response (Fig. 3). When comparing the response thresholds of 5 and 5000 BAU/mL, no difference in risk of COVID-19 related death was found at 7, 14, or 21 days following infection. However, the hazard ratio at 40 and 60 days post infection was 1.71 (p = 0.0089) and 1.75 (p = 0.0037), respectively. Comparing the same response thresholds, the hazard ratio of ICU admission at 14 and 28 days following infection was 1.78 (p = 0.0074) and 1.79 (p = 0.0014), respectively (Figure S4).

Of the 74 KTRs experiencing COVID-19 related deaths, 23 (31%) were administered post-exposure therapeutic IgG anti-RBD monoclonal antibodies after hospital admission (casirivimab/imdevimab, sotrovimab, or cilgavimab/tiksagevimab). Before receiving such biologics, this cohort had a median [IQR] anti-RBD IgG of 2 [2–5] BAU/mL. In general, mycophenolic acid was reduced to 250 mg or 500 mg BID following proven infection with SARS-CoV-2, and paused for 5–8 days in hospitalised patients. CNI was not withheld, but trough-targets were lowered.

### Effect of SARS-CoV-2 infection on excess mortality

**Fig. 4:**
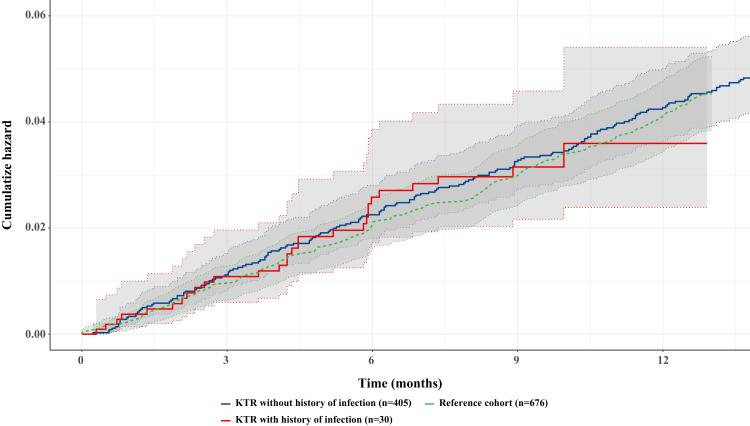
**(Risk of death not attributable to COVID-19)**: Cumulative hazard for death not related to COVID-19 in patients with (red) and without (blue) history of infection with SARS-CoV-2 during the pandemic, and a reference cohort from 2019 (green). Abbreviations: KTR, kidney transplant recipient.

The hazard of death both unrelated and related to COVID-19 one year after state entry, i.e. one year after the start of the pandemic for those not infected and one year from date of infection for COVID-19 survivors were 0.036 [95% CI: 0.024, 0.054] and 0.043 [95% CI: 0.036, 0.050] for KTRs with and without history of infection, respectively (Fig. 4), and these hazards were not different from the one-year hazard of death in the 2019 cohort (0.041 [95% CI: 0.034, 0.048]). Patients receiving an organ from a living donor had lower risk of COVID-19 related death (Table 1). While the risk of infection with SARS-CoV-2 was lower in elderly patients, age above 70 was associated with a greater risk of COVID-19 related death (Table 1). KTR who died due to COVID-19 were older and had more often received an organ from a deceased donor when compared to KTR survivors (median age of 71 [62, 76] vs 54 [45, 64] years and 74% (55/74) vs 59% (605/1020) with an organ from a deceased donor). Additionally, risk of infection was slightly greater in patients of female sex (Table 1).

**Table 1:** Effect of covariates on the competing risk of infection, COVID-19 related and unrelated death.

Event	Covariate	HR (95% CI)
Infection with SARS-CoV-2 n = 1094	Living donor (compared with deceased)	1.03 (0.91, 1.16, p = 0.63)
	Female sex (compared with male)	**1**.**15 (1.02, 1.30, p** = **0.025)**
	Age >70 years (compared with age <70 years)	**0**.**67 (0.57, 0.79, p < 0.0001)**
	Time after Tx < 2 years (compared with >2 years)	1.16 (0.98, 1.37, p = 0.081)
Death (non-COVID-19) n = 405	Living donor (compared with deceased)	**0.74 (0.60, 0.92, p** = **0.0068)**
	Female sex (compared with male)	0.96 (0.78, 1.17, p = 0.67)
	Age >70 years (compared with age <70 years)	**4.58 (3.76, 5.58, p < 0.0001)**
	Time after Tx < 2 years (compared with >2 years)	**0.54 (0.37, 0.79, p** = **0.0016)**
COVID-19 related death n = 74	Living donor (compared with deceased)	**0.53 (0.31, 0.90, p** = **0.018)**
	Female sex (compared with male)	1.07 (0.66, 1.71, p = 0.79)
	Age >70 years (compared with age <70 years)	**6.22 (3.93, 9.84, p < 0.0001)**
	Time after Tx < 2 years (compared with >2 years)	0.60 (0.28, 1.32, p = 0.21)
Death (non-COVID-19) after infection n = 30	Living donor (compared with deceased)	**0.22 (0.08, 0.64, p** = **0.0054)**
	Female sex (compared with male)	1.16 (0.56, 2.42, p = 0.68)
	Age >70 years (compared with age <70 years)	**6.10 (2.96, 12.55, p < 0.0001)**
	Time after Tx < 2 years (compared with >2 years)	0.34 (0.08, 1.44, p = 0.14)

Data is presented as hazard ratio (HR) with 95% confidence interval (95% CI). Values marked in bold are statistically significant to p < 0.05. Initial risk set consists of 3607 patients.

Prior to the social re-opening there were no differences in risk of non-COVID-19 related death in KTR without history of infection SARS-CoV-2 showing different vaccine responses. After the re-opening, the slope of the cumulative hazard function for death increased in patients with <5 BAU/mL compared with <5000 BAU/mL (Figure S5). At 30 months after the start of the pandemic, the cumulative hazard of non-COVID-19 related death in non-responders was 0.16 [95% CI: 0.14, 0.18] and 0.13 [95% CI: 0.11, 0.14] for the thresholds of ≥5 and ≥ 5000 BAU/mL, respectively.

## Discussion

In this nationwide cohort we found that the level of humoral vaccine response, as measured by anti-RBD BAU/mL, in KTR was predictive of survival following infection with the Omicron variant of SARS-CoV-2. Importantly, there were no differences between different thresholds of vaccine response in survival the first three weeks after infection, but high anti-RBD IgG titer response prior to infection was predictive of protection from day 40 after infection. Impaired vaccine response was also predictive of a reduction in admission to the ICU from day 14 after infection. This indicates that vaccine response protects against severe infections in the acute phase, and prevents fatal disease progression.

We cannot conclude that protection against COVID-19 related death was mediated by anti-RBD antibodies. Rather, the antibody titer may serve as a clinically useful biomarker for the global vaccine response, also including cellular responses, which may be modulated by a multitude of factors such as immunosuppression, age, and other co-morbidities. Anti-RBD IgG has previously been found to be associated with neutralising activity, both against BA.4/BA.5 in patients undergoing hemodialysis and against SARS-CoV-2 pseudo-virus in KTRs, which our findings appear to corroborate.^28^^,^^29^ Considering the present results demonstrating the usefulness of measuring anti-RBD titers in KTR, we suggest routine surveillance measurements following vaccination. Such measurements will allow for individual recommendations for booster doses, social distancing, and/or the need for pre-exposure prophylaxis, i.e. possibly new monoclonal antibodies.

In this KTR population, the risk of non-COVID related death was not increased following infection with SARS-CoV-2. Additionally, the risk of death was not different during the pandemic when compared with the previous year. This is an important finding, as it demonstrates that even though the country was under strict social restrictions, adequate care for this vulnerable patient population was provided. However, following social re-opening of the country, risk of non-COVID-19 related death increased slightly, and more in patients unable to produce humoral vaccine response ≥5 BAU/mL when compared with ≥5000 BAU/mL. A possible explanation is that non-responders may have an underlying cause for reduced vaccine response, which leaves them more susceptible to other infections. Elderly KTRs, who are known to have an impaired vaccine response, demonstrated a reduced risk of infection compared to their younger counterparts. This may be due to higher degree of adherence to social restriction, but likely also due to the fact that many may have been retired form the workforce, with reduced exposure from the workplace and from commuting.

The Norwegian population of KTRs experienced relatively low infection rates during the early phase of the pandemic, likely due to the rigid adherence to social restrictions, which we have previously demonstrated.^2^ However, following introduction of the Omicron variant infection rates among KTRs increased to levels comparable with the general Norwegian and European population. As such, KTRs in Norway were similarly exposed, allowing for generalisation of the data to other European countries.

There are some important limitations in this work. As vaccines were only available for a short duration of the Alpha, Beta, and to some extent the Delta variant periods, when vaccine responses were generally lower due to the very limited number of doses administered, our results should be considered mainly informative for the Omicron variant. Additionally, it is not possible to separate the effect of calendar time from that of increased vaccine response, as they are co-variant as vaccine responses developed with time. Most included patients were Caucasians, and one should therefore be careful to generalise our findings to other ethnicities.

A major strength of the present work is the high quality of the available data, with no patients lost to follow-up. Data were reported by the treating nephrologist, and underwent an initial quality control before data were added to the NRR, providing additional level of data quality compared to usual registry-based studies. However, we did not consider re-infections or the effect of breakthrough infection on seroconversion, due to the relatively low number of confirmed cases. The majority of included patients were on triple immunosuppressive maintenance therapy containing CNI, mycophenolic acid and prednisolone. This is currently the most common regimen used following kidney transplantation, but it is important to keep in mind that other drug combinations may have a different effect on vaccine response.

Kidney transplant recipients demonstrate low serological conversion rates following vaccination against SARS-CoV-2. While we found no differences in risk of infection between different levels of anti-RBD IgG concentrations, the risk of ICU admission and COVID-19 related death was lower in patients capable of producing greater humoral vaccine response. Additionally, no excess mortality was seen in KTRs with history of SARS-CoV-2 infection compared to a pre-pandemic reference cohort from 2019.

## Contributors

M.H., A.Å., L.A.M., J.T.V., F.L-J., K.M. conceptualised the research. A.Å., A.V.R., K.H., F.L-J., K.M. curated the data. M.H., A.Å. performed the formal analysis. L.A.M. performed funding acquisition. A.Å., A.V.R., K.H., F.L-J., K.M. performed the investigation. M.H., A.Å. determined the methodology. A.Å., L.A.M., J.T.V., F.L-J., K.M. administered the project. A.Å., L.A.M., J.T.V., F.L-J., K.M. provided the necessary resources. M.H., A.Å. developed the necessary software and scripts. A.Å. performed supervision. M.H., A.Å. provided visualisation. M.H., A.Å. wrote the original draft. M.H., A.Å., L.A.M., K.H., A.V.R., J.T.V., F.L-J., K.M. reviewed and edited the manuscript. All authors had full access to the data, and accept responsibility for the decision to submit for publication. M.H., A.Å. and F.L-J. have verified the underlying data.

## Data sharing statement

Data are available upon e-mail request to the corresponding author, provided that the proposed use of data is according to the consent given by the participants and Norwegian laws and legislations. If the criteria are fulfilled, de -identified individual data may be provided.

## Declaration of interests

A.Å. has received a speaker fee from Orifarm and travel support from Steiner. L.A.M. has received a fee for expert testimony from the Norwegian Medicines Agency. M.H., K.H., A.V.R., J.T.V., F.L-J., and K.M. declare no competing interests.
