# Supplementary material for: Humoral vaccine response and breakthrough infections in kidney transplant recipients during the COVID-19 pandemic: a nationwide cohort study
Source: eClinicalMedicine. 2023 Jun 6;60:102035. doi: 10.1016/j.eclinm.2023.102035 (PMC10242148; doi:10.1016/j.eclinm.2023.102035)
Supplement: Supplementary material [file mmc1.pdf]

SUPPLEMENTARY DATA

Table of Contents

Figure S1 – Page 2
Figure S2 – Page 2
Figure S3 – Page 3
Figure S4 – Page 3
Figure S5 – Page 4

Table S1 – Page 5
Table S2 – Page 5

Study Protocol
STROBE checklist

Humoral vaccine response and breakthrough infections in kidney transplant recipients during the COVID-19 pandemic: a nationwide cohort study

Markus Hovd, MSc. Pharm.[1,2,3], Anders Åsberg, PhD[1,2,3], Ludvig A. Munthe, PhD[4,5], Kristian Heldal, PhD[1,6], Anna V. Reisæter, PhD[1,3], John T. Vaage, PhD[4,7], Fridtjof Lund-Johansen, PhD[7,8], Karsten Midtvedt, PhD[1]

[1] Department of Transplantation Medicine, Oslo University Hospital, Norway
[2] Department of Pharmacy, University of Oslo, Norway
[3] The Norwegian Renal Registry, Department of Transplantation Medicine, Oslo University Hospital, Norway.
[4] Institute of Clinical Medicine, University of Oslo, Norway
[5] KG Jebsen Centre for B cell Malignancies, Institute of Clinical Medicine, University of Oslo, Norway
[6] Institute of Health and Society, University of Oslo, Norway
[7] Department of Immunology, Oslo University Hospital, Norway
[8] ImmunoLingo Convergence Center, Institute of Clinical Medicine, University of Oslo, Norway

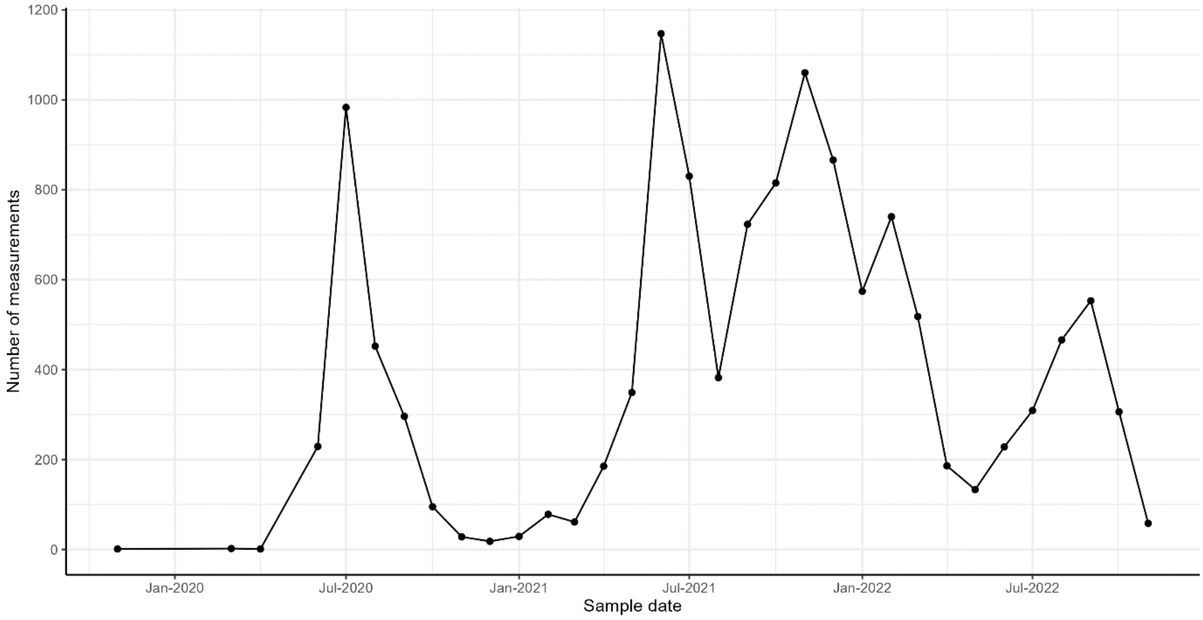

Figure S1: Overview of sample collection for quantification of anti-RBD IgG. Measurements prior to January 2021 were indicated for detecting breakthrough infections. Following introduction of vaccines (January 2021), measurements were made to quantify humoral vaccine response.

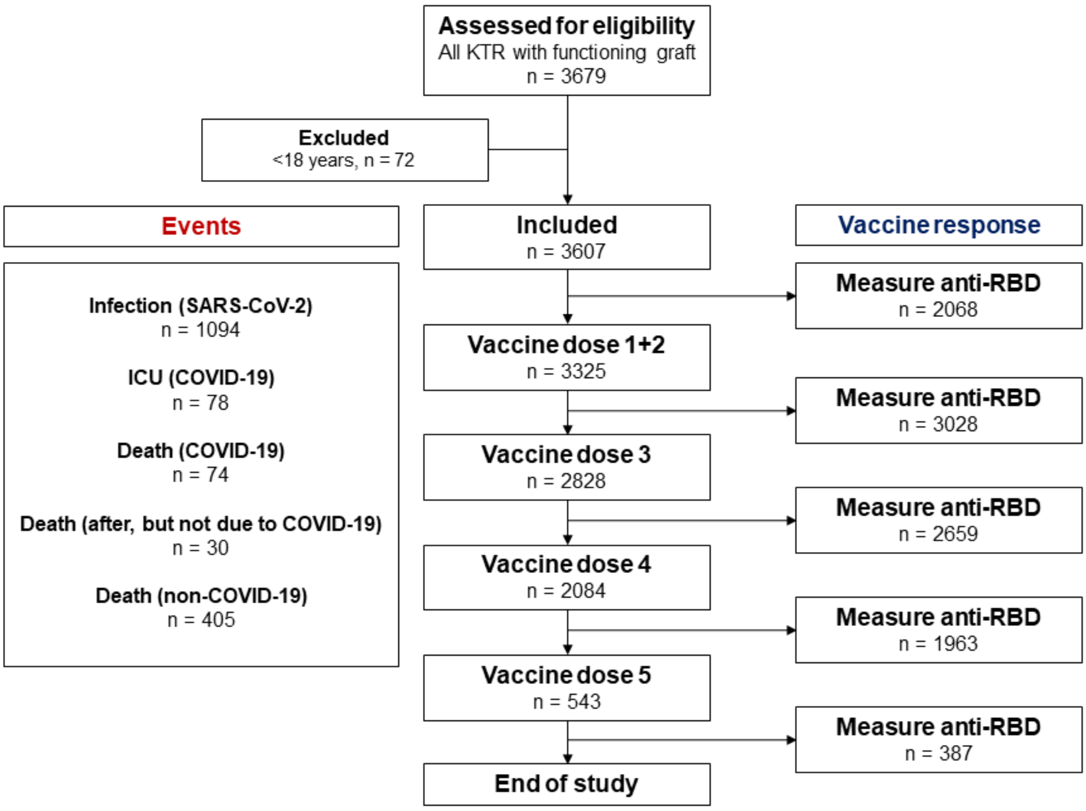

Figure S2: Flowchart detailing the inclusion and exclusion of patients, number of patients who received each vaccine dose, and the number of patients with anti-RBD measurements following vaccination.

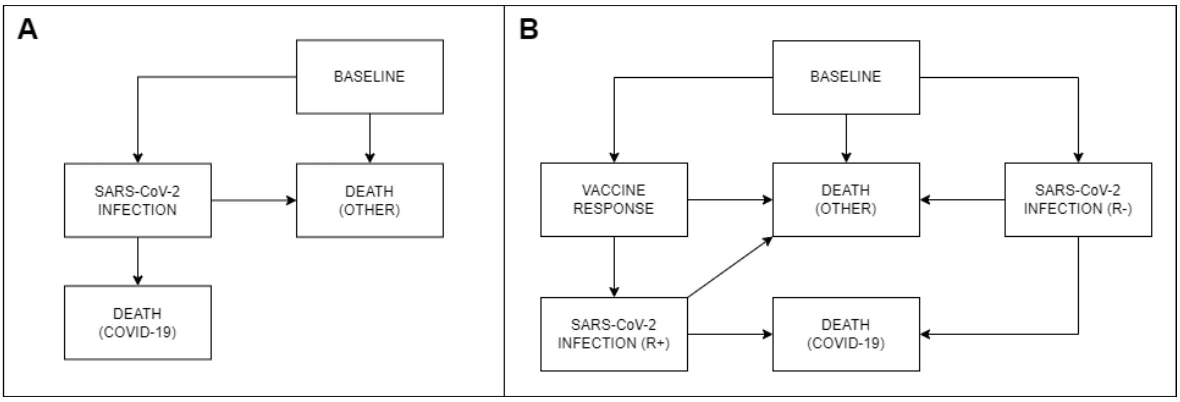

Figure S3: Structural overview of the A) basic and B) extended multi-state model. R(+) and R(-) refers to vaccine response and lack thereof, respectively, at the time of infection.

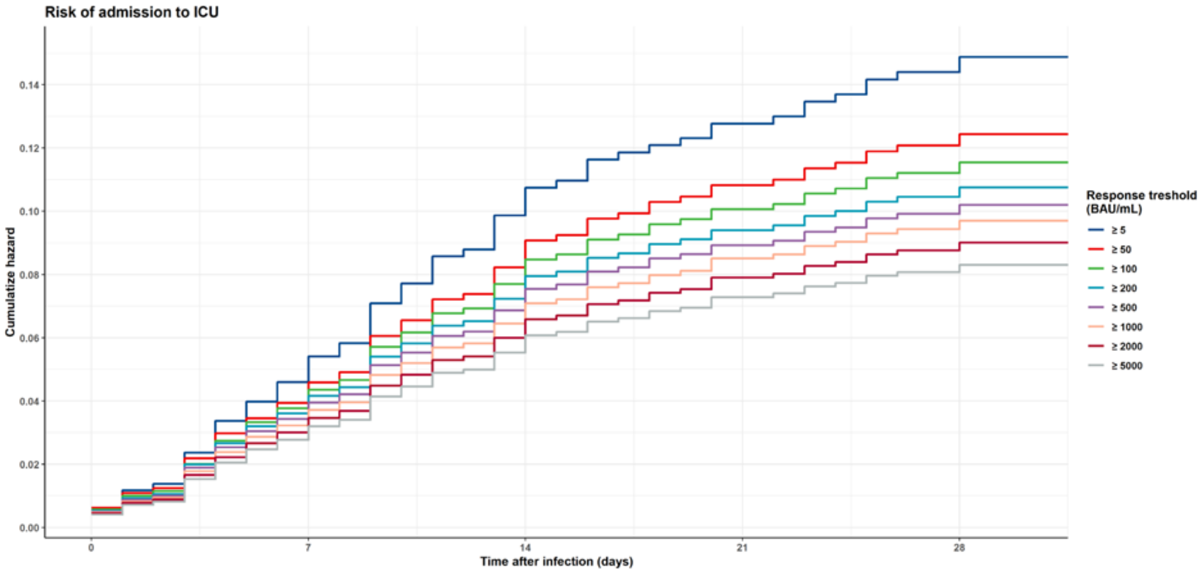

Figure S4: Risk of admission to ICU, in patients infected with SARS-CoV-2, from time of infection in non-responders (by vaccine response thresholds).

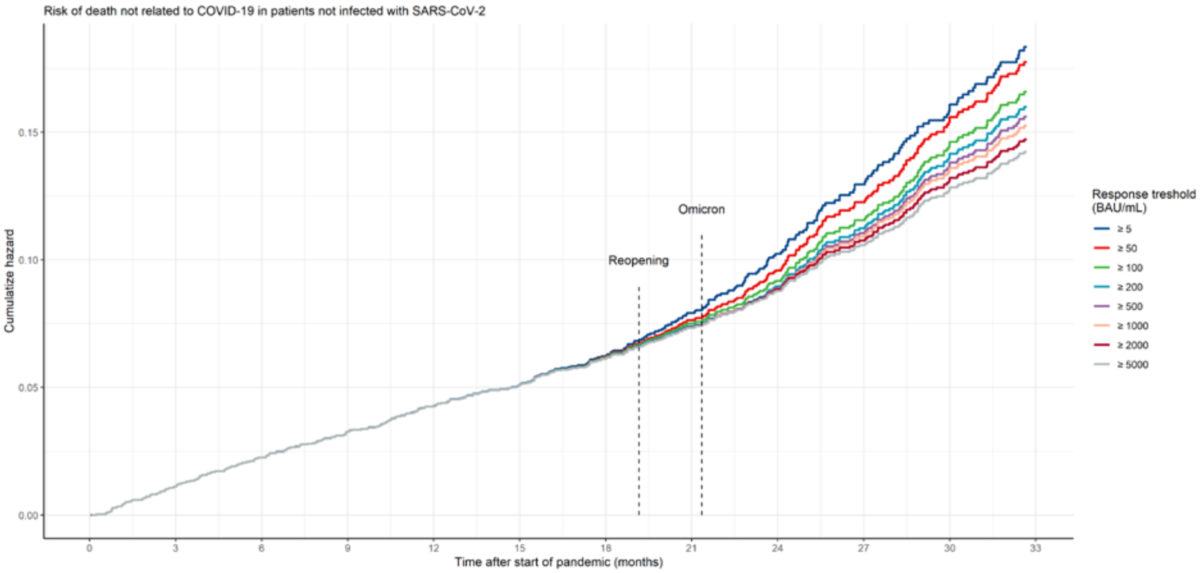

Figure S5: Risk of death not related to COVID-19, in patients not infected with SARS-CoV-2, since the start of the pandemic in non-responders (by vaccine response thresholds).

Table S1: Summary of immunosuppressive regimen in the main cohort.

Immunosuppressive regiment at start	Number of patients
TAC + MPA + PRED	2089
CSA + MPA + PRED	644
CSA + PRED	205
TAC + PRED	184
MPA + PRED	149
CSA + AZA + PRED	92
TAC + AZA +PRED	55
Other	169
Uknown / none	20

TAC: tacrolimus, MPA: mycophenolic acid, PRED: prednisolone, CSA: cyclosporine A

Table S2: Summary of vaccination time and manufacturer for the initial vaccination and booster doses.

	Vaccination time (months [80% quantile] after start of pandemic)	Number vaccinated† (n, %)	Manufacturer		
			Pfizer	Moderna	Other††
Dose 1+2	14 [13, 15]	3325 (92%)	6163 (93%)	465 (7%)	22 (0%)
Dose 3	19 [19, 20]	2828 (78%)	1803 (64%)	1025 (36%)	0 (0%)
Dose 4	23 [22, 23]	2084 (58%)	853 (41%)	1224 (59%)	7 (0%)
Dose 5	30 [29, 31]	543 (15%)	231 (43%)	294 (54%)	18 (3%)

† Percentage vaccinated do not account for death, breakthrough infection, or refusal to vaccinate.

†† Other vaccine manufacturers include Vaxzevira® (Astra-Zeneca), Nuvaxovid® (Novovax) and Jcovden® (Johnson & Johnson).

SARS-CoV-2 immune response in kidney transplant recipients and dialysis patients

[SARS-CoV-2 IgG-KTx-study]

Principal investigator:

Karsten Midtvedt, MD, Ph. Department of Transplant Medicine, Oslo University Hospital – Rikshospitalet, Oslo, Norway.

Scientific collaborators:

Fridtjof Lund-Johannsen, MD PhD Department of Immunology, OUS-RH
John Torgils Vaage, Professor PhD Department of Immunology, OUS-RH
Grete B Kro, MD PhD, Department of Microbiology, OUS-RH

Anders Åsberg, Professor, Ph.D., Department of Transplant Medicine, OUS-RH.

Krystina Parker, overlege, AHUS
Elisabeth H. Kvien, overlege, Arendal Sykehus
Hans Petter Marti, Professor, Haukeland Universitetssykehus
Camilla Madsen, overlege, Bodø Sykehus
Tale N. Wien, overlege, Bærum Sykehus
Morten R. Nielsen, overlege, Drammen Sykehus
Stine Volden, overlege, Elverum Sykehus
Viera Stubnova, overlege, Finnmarksykehuset
Astrid Dale, overlege, Førde Sykehus
Øystein Nymoen, Harstad Sykehus
Tor H. Quale, overelege, Haugesund Sykehus
Jan Bitter, overlege, Kristiansand Sykehus
Solfrid Romunstad, overlege, Levanger Sykehus
Kari Mørkve Soldal, overlege, Lillehammer Sykehus
Geir Norbø, overlege, Ringerike Sykehus
Lars Ulrik Broch, overlege, Skien Sykehus
Lasse Gøransson, overlege, Stavanger Universitetssykehus
Monica Christiansen, overlege, Stord Sykehus
Marit Solbu, overlege, Tromsø Universitetssykehus
Cicilia M. Øien, overlege, St. Olavs Hospital, Trondheim
Stig Arne Kjellevold, overlege, Vestfold Sentralsykehus, Tønsberg

Aud-Eldrid Stenehjem, Overlege, Ullevål Universitetssykehus
Gro Lillegraven, overlege, Voss Sykehus
Julia Smedbråten, overlege, Østfold Sykehus, Fredrikstad
Anne- Beathe Tafjord, overlege, Ålesund Sykehus

Background

Following a kidney transplantation (KTx) patients are in need of life-long immunosuppressive drug treatment. Immunosuppression increases the risk of getting contagious infective diseases. It has therefore been anticipated that solid organ transplant (SOT) patients have been very vulnerable during the ongoing SARS-CoV-2 pandemic. Currently there is, however, limited information on how SARS-CoV-2 has affected the SOT population. There is an ongoing European study lead by European Renal Association (ERA-EDTA) collecting prospective data on outcome from kidney transplant (KTx) and dialysis patients whom have tested positive for SARS-CoV-2 in a nasopharynx swab test (REK # 125548 COVID-19 utfall i pasienter med kronsik nyresykdom). The Norwegian Renal Registry (NRR) is prospectively reporting to this European data-base.

The presentations of symptoms during a SARS-CoV-2 infection are many. For most affected patients the primary symptoms have been from the upper respiratory tract with dyspnea, fever and coughing, initially rather like the common flue. Eventually there have been reports on many different symptoms associated with a SARS-CoV-2 infection (GI problems, loss of taste/smell, confusion, muscular pain, fatigue etc). Due to limited test capacity persons tested, so far, for possible SARS-CoV-2 have predominantly had upper respiratory symptoms.

Currently a blood test to see if you have had SARS-CoV-2 infection is being established in Norway, at Oslo University Hospital – Rikshospitalet (OUS-RH). This test will detect the presents of an antibody (IgG) against the virus produced by the B-cells; SARS-CoV-2 IgG. Normally, detection of an anti-virus IgG antibody means that you are immune towards this virus, at least for some time. Whether this also is the case following SARS-CoV-2 infection has not been fully evaluated. In order to produce SARS-CoV-2 IgG the immune system must

have been exposed to the virus and also have the capacity to respond. Use of immunosuppression, i.e. like after KTx, is known to affect a patient's immune system and maybe also the capacity to respond properly with IgG production towards SARS-CoV-2. Currently there is a lack of knowledge regarding KTx patients and their ability of developing SARS-CoV-2-IgG. If they develop SARS-CoV-2 IgG, how long does it last? Is it protective?

It has also been speculated on the fact that some of the severely ill patients in ICU's seem to have had an "over-reactive" immune response creating a treatment-challenge when patients were in need of ventilators, dialysis etc. Some authors anticipate that some immunosuppressed patients actually may have had less severe symptoms, i.e. be infected with SARS-CoV-2 but not really feeling ill.

The Norwegian Renal Registry (NRR), a consent based national medical quality register, has been collecting data on patients started on renal replacement therapy for more than 30 years. The written informed consent allows the registry to keep track of the patients, their treatment modality and collect annual data on treatment relevant variables and medical events life-long. The data can be used for research and quality analyses following individual project approval from REK. There are 26 centers responsible for reporting data on an annual basis to NRR. Currently the individual coverage is close to 100% (36 patients alive without consent). The same centers are now reporting data on all positive SARS-CoV-2 KTx and dialysis patients in Norway (REK # 125548), currently representing a cohort of approximately 3,700 patients with a functioning kidney graft and 1,700 patients in dialysis. Data gathered so far by NRR show that (at this date) 37 patients with a function renal graft and 27 in dialysis have presented a positive SARS-CoV-2 nasopharynx test and 7 (18.9%) and 4 (14.8%), respectively, have died from COVID-19 in Norway. The numbers are expected to increase. The cooperation between

the national transplant center and the 26 local nephrology units is unique and gives Norway a possibility to get national data on the entire KTx cohort regarding development of SARS-CoV-2 IgG. It also gives Norway a unique opportunity to get data on the KTx and dialysis cohort regarding development of post-vaccination SARS-CoV-2 IgG.

Rationale

Immunosuppressed (e.g. kidney transplanted patients) and immunocompromised (e.g. patients in dialysis) patients do not necessarily respond adequately to SARS-CoV-2 infection with production of IgG. If they produce SARS-CoV-2 IgG it is unknown how long the response last. Since proper immune reactions against the virus is needed in order to be immune, in-depth knowledge about these questions are needed in order to provide appropriate treatment of this vulnerable cohort of patients. It will probably be possible to extrapolate knowledge from kidney transplant recipients to other immunosuppressed patient groups. It is now possible to measure SARS-CoV-2 IgG in an ordinary blood samples and with total overview of the entire Norwegian kidney transplant cohort in NRR and the well-functioning cooperation between the national transplant center and local nephrology units it is possible to obtain the necessary blood sample from all patients within a 3-month period, in time before the second COVID-19 wave.

Immunosuppressed/-compromised patients do not necessarily respond adequately to SARS-CoV2 vaccines either. No solid organ transplant recipients or dialysis patient were included in any of the published trials from the currently EMA approved SARS-CoV2 vaccines (Pfizer/BioNTec and Moderna) nor in the soon to be approved vaccine from AstraZeneca/Oxford. According to current knowledge the mortality rate for kidney transplanted and hemodialysis patients from COVID-19 is appr. 20 % in Norway and Europe

[1]. Internationally mortality rate during omicron wave 1 and 2 have been found to be approximately 5%. Similar findings are anticipated for the Norwegian kidney transplant population. Norwegian Institute of Public Health strongly recommends immunosuppressed patients and patients in hemodialysis to be vaccinated and now to also receive booster doses. NRR has coverage of close to 100% for both transplanted recipients and patients in dialysis. ~~Measurement of a post-vaccine SARS-CoV2 Spike IgG is possible.~~ We have followed initial vaccine response in kidney transplanted immunocompromised patients. It has become clear that they are in need of additional booster vaccinations. Most of our patients have now received several SARS-CoV-2 booster doses. International data has shown that after 3 or 4 vaccines approximately 30-40 % of kidney transplant recipients still have no adequate vaccine induced IgG SARS-CoV-2 antibody response. In responders it looks like this response only last for 4-5 months. Therefore, we wish to continue to offer the possibility to monitor SARS-CoV-2 IgG in the kidney transplant recipients.

Ethical considerations

The project needs one blood sample from every participant following each vaccination. On a routinely basis KTx recipients have life-long check-ups, including blood-tests, every 3 months. This sample can therefore be obtained when patients are donating blood in a standard clinical situation, i.e. there is general no need for extra visits or extra venopunctures and hence no extra burden for the patients. Hence, we do not believe there will be any risk for the patients.

Study objectives

The objectives are to get increased knowledge on development of SARS-CoV-2 IgG in a KTx population and how this is related to clinical signs of COVID-19 (i.e. if they have presented themselves to a hospital for COVID-19 signs or not).
To validate if SARS-CoV-2 IgG lasts in KTx recipients and to validate the analytic method in immunocompromised patients.
In addition to get increased knowledge on the development of SARS-CoV-2 Spike IgG following vaccination.

Questions that will be answered by this study;

- Do KTx recipients with known exposure to SARS-CoV-2 develop SARS-CoV-2 IgG?

- What is the current percentage of KTx patients that have had SARS-CoV-2 without being sick/contacting their physician?

- Will SARS-CoV-2 IgG be protective during the next "wave" of the pandemic?

- Do the currently approved SARS-CoV-2 vaccines act equally well on immunosuppressed kidney transplant recipient and in patients requiring dialysis?

Study design

The study team at OUS-RH will prepare and fill-in blood-requisitions for a SARS-CoV-2 IgG test for all patients whom previously have agreed to be registered in the NRR. These requisitions will be mailed directly to the participant along with a study information letter informing the participant about this study. By taking the blood-test they agree to participate in the study. Many patients have already (on their personal initiative) copied previous blood-requisitions and taken a new test after a vaccine booster dose. The local nephrologists at the 26

centers who follow the respective recipient locally will receive the answer from the SARS-CoV-2 IgG test directly, with a copy to the study team at OUS-RH.

The study team at OUS-RH will prepare and fill-in blood-requisitions for a SARS-CoV-2 IgG test (to be taken 4-10 weeks after vaccine each booster dose) for all patients whom previously have agreed to be registered in the NRR. These post-vaccination requisitions will be mailed directly to the patients along with a new study information letter informing the participants about the study. By taking the blood-test they agree to participate in the study. The local nephrologists at the 26 centers who follow the respective recipient/dialysis patients locally will receive the answer from the SARS-CoV-2 IgG test directly, with a copy to the study team at OUS-RH.

If KTx patients or dialysis patients have additional questions they may ask their local nephrologist/name-given nephrologist at OUS-RH (details included in the study information letter all patients receive). If they want to participate, they take the blood test at the next check-up at the hospital or at their local private practitioner etc).

The local nephrologist will inform the patients about the SARS-CoV-2 IgG result. If positive the patients are contacted directly, if negative the patients will get the information at the next standard follow-up visit. Patients eager to know the result may contact their local nephrologist also before the next standard follow-up visit. This is also outlined in the information letter.

The local nephrologist will inform the patients about the post-vaccination SARS-CoV-2 IgG result during the next out-patient visit (regularly 4 times /year in KTx patients) or when available in dialysis patients (whom are in the hospital 3/week (hemodialysis) or at least 4

times/year (peritonealdialysis)). We have organized a “vaccine-hot-line/cell-phone” which has been operating throughout the pandemic. All patients are free to call if they have questions/wonder about their individual vaccine response.

Inclusion criteria

- Patients transplanted with a kidney (only).
- Patients in dialysis
- Age of 18 years or older.
- Previously sign informed consent to be registered in the NRR

Exclusion criteria

- None

Patients

Adult kidney transplanted patients on a diversity of different immunosuppressive drugs and that have previously signed informed consent to be registered in NRR are eligible for inclusion in the project.

Study procedures

Every patient registered in NRR will receive an envelope with a letter giving study information. Included in this study information there will be the name of a physician working at the patients regular check-up hospital in charge of the study and the name of a study contact

person at OUS-RH including telephone number. Along with the letter there will be a pre-filled blood-requisition for SARS-CoV-2 IgG or post-vaccination SARS-CoV-2 IgG. If the patient agrees to participate, she/he needs to take the blood sample. This blood sample can be taken at any local hospital or private practitioner in Norway. The answer will go directly to the local contact nephrologist. If SARS-CoV-2 IgG is positive the participant will be contacted by their local physician. If SARS-CoV-2 IgG is negative the patient will be informed at next regular check-up. Result from the post-vaccine SARS-CoV-2 IgG will be given at next regular check-up.

Withdrawals and premature study termination

Patients are free to withdraw their consent to this project at any time and this will not impact their further treatment.

Statistical methodology

Results will be tabulated descriptively.

Number of patients

Not applicable. The aim is to get a complete national overview of the kidney transplant cohort so all will be invited and we anticipate that a minimum of 70-80% donate a blood sample to the study. Specific interest will be addressed toward known previously SARS-CoV-2 naso-pharynx positive KTx recipients still alive.

Analysis plan

Blood samples will be analyzed consecutively as they arrive at OUS-RH and answered directly to the treating physician, with a copy to the study team at OUS-RH, without further delay.

Time Schedule

Study start: Q2, 2020

End of study: Anticipated end of study is Q2 2025.

Subject confidentiality

The subject has a right for a protection against invasion of privacy. The validation and clinical applicability part of the prospective registration will have to be performed with fully identified samples.

Representatives from any Regulatory Authority, as well as the appropriate Ethical Committee are permitted to review the subject's primary medical records including laboratory test result reports, admission and discharge summaries if needed.

Publication plan

The results from the analyses will be presented at national and international congresses as well as published in international peer reviewed journals. All data will be presented unidentifiable so no data can be linked to a specific patient. The Principal Investigator is responsible to catalyze the writing of the different publications and in cooperation with the collaborators

involved collectively decide which publications to be produced and to find a responsible writer for each publication.

Financial issues

Each participating partner will provide internal financial resources and labor into the study.

Quality assurance and approvals

The study will be performed according to the Declaration of Helsinki. The study has been evaluated by the Regional Committee for Medical Research ethics, Health region southeast prior to study start.

Insurance

Standard insurance in NPE (Norsk Pasientskade Erstatning).

References

1. Hilbrands, L.B., et al., *COVID-19-related mortality in kidney transplant and dialysis patients: results of the ERACODA collaboration.* Nephrol Dial Transplant, 2020. **35**(11): p. 1973-1983.

STROBE Statement—Checklist of items that should be included in reports of ***cohort studies***

	Item No	Recommendation	Page No
**Title and abstract**	1	(*a*) Indicate the study's design with a commonly used term in the title or the abstract<br>(*b*) Provide in the abstract an informative and balanced summary of what was done and what was found	Page 1, Title<br>Page 2, Abstract
**Introduction**			
Background/rationale	2	Explain the scientific background and rationale for the investigation being reported	Page 2 (Research in context)
Objectives	3	State specific objectives, including any prespecified hypotheses	Page 3, Introduction, last paragraph
**Methods**			
Study design	4	Present key elements of study design early in the paper	Page 3-4, Methods
Setting	5	Describe the setting, locations, and relevant dates, including periods of recruitment, exposure, follow-up, and data collection	Page 3-4, Methods
Participants	6	(*a*) Give the eligibility criteria, and the sources and methods of selection of participants. Describe methods of follow-up<br>(*b*) For matched studies, give matching criteria and number of exposed and unexposed	Page 3-4, Methods<br>Not relevant, not a matched study
Variables	7	Clearly define all outcomes, exposures, predictors, potential confounders, and effect modifiers. Give diagnostic criteria, if applicable	Page 3-4, Methods.
Data sources/ measurement	8*	For each variable of interest, give sources of data and details of methods of assessment (measurement). Describe comparability of assessment methods if there is more than one group	Page 3-4, Methods
Bias	9	Describe any efforts to address potential sources of bias	Not relevant
Study size	10	Explain how the study size was arrived at	Not relevant
Quantitative variables	11	Explain how quantitative variables were handled in the analyses. If applicable, describe which groupings were chosen and why	Page 3-4, Methods. No grouping.
Statistical methods	12	(*a*) Describe all statistical methods, including those used to control for confounding<br>(*b*) Describe any methods used to examine subgroups and interactions<br>(*c*) Explain how missing data were addressed<br>(*d*) If applicable, explain how loss to follow-up was addressed<br>(*e*) Describe any sensitivity analyses	a) Page 4, Methods<br>b) Not relevant<br>c) No missing data<br>d) No loss to follow-up<br>e) No sensitivity analysis performed
**Results**			
Participants	13*	(a) Report numbers of individuals at each stage of study—eg numbers potentially eligible, examined for eligibility, confirmed eligible, included in the study, completing follow-up, and analysed<br>(b) Give reasons for non-participation at each stage<br>(c) Consider use of a flow diagram	a) Page 4-5, Results and Table 1<br>b) Not relevant<br>c)
Descriptive data	14*	(a) Give characteristics of study participants (eg demographic, clinical, social) and information on exposures and potential confounders	a) Page 4-5, Results.

		(b) Indicate number of participants with missing data for each variable of interest	b) No missing data
		(c) Summarise follow-up time (eg, average and total amount)	c) Page 4-5, Results
Outcome data	15*	Report numbers of outcome events or summary measures over time	Page 4-5, Results and Table 1

Main results	16	(*a*) Give unadjusted estimates and, if applicable, confounder-adjusted estimates and their precision (eg, 95% confidence interval). Make clear which confounders were adjusted for and why they were included	a) Unadjusted estimates provided in Page 4-5, Results. Covariate-adjusted estimates provided in Table 1
		(*b*) Report category boundaries when continuous variables were categorized	b) Page 3-4, Methods
		(*c*) If relevant, consider translating estimates of relative risk into absolute risk for a meaningful time period	c) Page 4-5, Results
Other analyses	17	Report other analyses done—eg analyses of subgroups and interactions, and sensitivity analyses	Not relevant
**Discussion**			
Key results	18	Summarise key results with reference to study objectives	Page 5-6, Discussion
Limitations	19	Discuss limitations of the study, taking into account sources of potential bias or imprecision. Discuss both direction and magnitude of any potential bias	Page 5-6, Discussion
Interpretation	20	Give a cautious overall interpretation of results considering objectives, limitations, multiplicity of analyses, results from similar studies, and other relevant evidence	Page 5-6, Discussion
Generalisability	21	Discuss the generalisability (external validity) of the study results	Page 6, Discussion
**Other information**			
Funding	22	Give the source of funding and the role of the funders for the present study and, if applicable, for the original study on which the present article is based	Page 2, Funding and Page 7, Declaration of interest

*Give information separately for exposed and unexposed groups.

Note: An Explanation and Elaboration article discusses each checklist item and gives methodological background and published examples of transparent reporting. The STROBE checklist is best used in conjunction with this article (freely available on the Web sites of PLoS Medicine at http://www.plosmedicine.org/, Annals of Internal Medicine at http://www.annals.org/, and Epidemiology at http://www.epidem.com/). Information on the STROBE Initiative is available at http://www.strobe-statement.org.
